# Defining Patterns and Rates of Natural vs. Drought Driven Aquatic Community Variability Indicates the Ongoing Need for Long Term Ecological Research

**DOI:** 10.3390/biology12040590

**Published:** 2023-04-12

**Authors:** Ivana Pozojević, Valentina Dorić, Marko Miliša, Ivančica Ternjej, Marija Ivković

**Affiliations:** Division of Zoology, Faculty of Science, University of Zagreb, Rooseveltov trg 6, 10000 Zagreb, Croatia; valentina.doric@biol.pmf.hr (V.D.); marko.milisa@biol.pmf.hr (M.M.); ivancica.ternjej@biol.pmf.hr (I.T.)

**Keywords:** community change, insect emergence, dipteran community, directional change, drought

## Abstract

**Simple Summary:**

Long-term research tends to fall short in the fast-track “publish or perish” research era, even in ecology where it is proven time and time again that natural community variability is sometimes hard to distinguish from the community’s response to environmental stressors. In this research, we present how unimpacted freshwater habitats and the dipteran communities therein deal with changing climate and discharge conditions with regard to community structure. Distinguishing “normal” from “extreme” events and taxa indicative of these periods was made possible only after analyzing long-term data. This research presents how freshwater indicator taxa and local dipteran diversity change significantly with regard to extremes in discharge regimes.

**Abstract:**

Most ecologists have used climate change, as an omnipresent pressure, to support their findings in researching the vulnerability of specific taxa, communities, or ecosystems. However, there is a widespread lack of long-term biological, biocoenological, or community data of periods longer than several years to ascertain patterns as to how climate change affects communities. Since the 1950s, southern Europe has faced an ongoing trend of drying and loss of precipitation. A 13-year research program in the Dinaric karst ecoregion of Croatia aimed to comprehensively track emergence patterns of freshwater insects (true flies: Diptera) in a pristine aquatic environment. Three sites, spring, upper, and lower tufa barriers (calcium carbonate barriers on a barrage lake system that act as natural damns), were sampled monthly over 154 months. This coincided with a severe drought event in 2011/2012. This was the most significant drought (very low precipitation rates for an extended period of time) in the Croatian Dinaric ecoregion since the start of detailed records in the early 20th century. Significant shifts in dipteran taxa occurrence were determined using indicator species analysis. Patterns of seasonal and yearly dynamics were presented as Euclidian distance metrics of similarity in true fly community composition compared at increasing time intervals, to ascertain the degree of temporal variability of similarity within the community of a specific site and to define patterns of similarity change over time. Analyses detected significant shifts in community structure linked to changes in discharge regimes, especially to the drought period.

## 1. Introduction

In recent decades, great efforts have been made to assess and mitigate the ecological impacts of anthropogenic pressures on aquatic ecosystems, with strong emphasis on stressors caused by climate change, which are ever-present and often difficult to quantify [1,2]. Assessing the impact of specific stressors is key if freshwater ecosystem conservation or impact-mitigation measures are to be achieved, yet assessments almost always fail to evaluate the impact of climate change. The present study aims to overcome interruptions by other stressors and the continuous-comprehensive research issues that arise when trying to determine the effects of climate change. This was conducted by using 13-year biological and ecological data on insect emergence patterns to examine the effects of climate change on an otherwise unimpacted freshwater ecosystem kept under a strict conservation regime for over seven decades, the perennial barrage lake system of the Plitvice Lakes National Park in Croatia [3,4,5,6,7,8,9,10,11,12,13].

In southern Europe, there is an overall drying trend in terms of the loss of precipitation volume occurring since the 1950s [14]. Climate modelling results for the early 21st century indicate a possible change in the precipitation volume throughout Europe, more specifically, precipitation loss in Croatia during the spring, summer, and autumn [15]. Within the study period, an extreme drought event occurred in 2011/2012. This was one of the most significant droughts since the early 20th century and the start of detailed records [16]. The drought started in February 2011 and lasted for 19 months [16]. The barrage lake system retained its continuous water flow throughout the drought event, despite a significant decline in water discharge into the system as compared to the earlier years of the study which were under stable climatological conditions. Flow or discharge reduction largely impacts the aquatic insect community, affecting functional groups such as filter feeders more than other groups (i.e., predators) [17]. Following such exceptional, non-seasonal low-flow episodes, community recovery usually takes several years [18], depending on its resistance and resilience [19].

The present study examines an omnipresent group that dominates the aquatic environment more than other freshwater organisms: true flies (Diptera) [20]. The true flies are known in environmental research as a widespread group, often supporting diverse communities in the food chain. Aside from serving as prey for other organisms, they are often well-adapted predators and play the role of ecosystem “cleaners” feeding on particulate organic matter. However, some taxa are also quite sensitive to environmental change and as such can serve as indicators of specific environmental conditions [20,21,22].

The study aimed to determine patterns and rates of natural vs. extreme flow-driven community variability with emerging aquatic true fly communities serving as models. We hypothesized that natural variability would be observed in the normal discharge period followed by community change influenced by extreme discharge regimes (especially during the drought period), and finally, possible community recovery in the normal discharge period. The goals were to (i) determine the response in the biological emergence data to the selected driver (explanatory variable), that is, water discharge as the environmental data most affected by drought, in order to portray the biological and environmental dynamics in three sites in the barrage lake system: spring, upper, and lower tufa barrier; (ii) compare the dissimilarity among communities at different time intervals (monthly and yearly samples) within each site to determine patterns of seasonal (cyclic) dynamics and directional change [23]; (iii) detect community change points that could be linked to extreme discharge periods; and (iv) determine dipteran taxa that were indicators of the normal, low, and high discharge periods at each site.

## 2. Materials and Methods

### 2.1. Study Area

**Figure 1:**
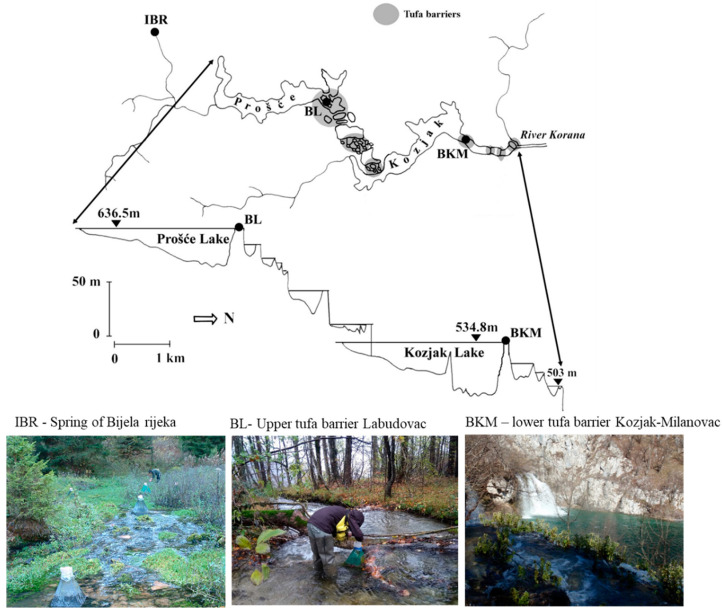
Map of the study area indicating the three sampling sites with photographs. IBR–Spring of the Bijela Rijeka River, BL–Upper (upstream) tufa barrier Labudovac, BKM–Lower (downstream) tufa barrier–Kozjak-Milanovac.

Dipteran communities were sampled at three perennial aquatic sites (longitudinally distributed habitats in a barrage-lake system) in Plitvice Lakes National Park (PLNP) in Croatia, located in the karst region of the north-western Dinarides mountain range (Figure 1). All necessary permits were obtained from the Ministry of Environment and Energy and the national park authorities. PLNP is a barrage-lake system, approximately 8.2 km in length, created by numerous tufa barriers featuring a high diversity of habitat types typical of karst systems, such as springs, streams, waterfalls, lakes, and tufa barriers [24]. These habitats support a high biodiversity of Diptera families. The climate is classified as Cfb according to the Köppen classification [25] and is specific as it is influenced by several neighboring climates: temperate and continental climates with warm summers but also by a boreal climate. The study sites were selected to encompass different habitats across a longitudinal gradient differing in environmental conditions in terms of discharge and water temperature.

### 2.2. Field Sampling and Laboratory Analysis

Dipteran individuals were collected on a monthly basis over a 13-year period from March 2007 to December 2019 (154 months) using six pyramid-type emergence traps at each site (154 months × 3 sites × 6 traps = 2772 samples). Each trap was a four-sided pyramid with a base of 45 × 45 cm and a height of 50 cm, covered with 1 mm mesh netting. Emergence traps were fastened to the streambed, with an unnetted gap of approximately 4 cm proximal to the streambed enabling the free movement of larvae both into and out of the sampling area. On top of each emergence trap was a collecting container filled with a preservative (2% formaldehyde with detergent as a surface-tension-reducing agent [26]). Traps were positioned in different representative microhabitats at each site, although the microhabitat structure is very dynamic and changeable over time (Table 1). After sampling, all specimens were counted and preserved in 80% ethanol. Taxonomic identification to the family level was based on [27,28].

Daily mean discharge data was obtained from the Croatian Meteorological and Hydrological Service from their three gauging stations located near the three sampling sites in the PLNP. Water temperature data were measured daily with the HOBO Pendant temperature data logger (#Part UA-001-XX, Bourne, MA, USA).

### 2.3. Statistical Analyses

Discharge data were tested for statistically significant differences among different years of the studied period for all three sites by performing a generalized mixed model (GLMM) with discharge as the response variable, years as the fixed effects, and months as random effects, after which extreme yearly discharges could be distinguished from normal yearly discharge rates [29]. The results of the GLMM are presented in the Supplementary Material. The relationship between water temperature and minimum, maximum, and average discharge was determined using Spearman’s correlation coefficient [29]. Monthly community composition (i.e., abundance values of each taxon) and values of water temperature and discharge were also plotted together, to depict the biological and environmental dynamics within the three sites along the longitudinal gradient. Prior to this testing, data were tested for normality using the Shapiro-Wilk test (Supplementary Material, SuppInfo_Diptera_Abundance).

Patterns of seasonal and yearly differences among sites were presented as Euclidian distance metrics in true fly community composition compared at increasing time lags set to monthly and yearly intervals, respectively. When the time lag was one (month or year), then distances were calculated for two consecutive monthly (or yearly) samples. If the time lag was two, then the distances were calculated between January and March, February and April (year 1 and 3, year 2 and 4… etc.), and so on. Time lags increase to a maximum of the time span between the first and last sample. The result is a triangular resemblance matrix containing Euclidian distances among true fly communities. Euclidian distance was used because of its wide range and clear geometric properties in similarity metrics, though other metrics such as Bray Curtis are also applicable. A Euclidian distance triangular resemblance matrix over time regression was done to determine the degree of temporal variability and the potential for clear patterns of change over time or alternatively, to show seasonal dynamics. There are three possible outcomes of the Euclidian distance/time lag regression: “*If the distance between samples does not change as time-lags increase, then the community is considered to be stable. If sample distance increases over time, the community is unstable and undergoing directional change. If sample distance decreases over time, then the community is unstable and undergoing convergence*” [23]. In this case, if the Euclidian distance triangular resemblance matrix over time regression does not show direction in the monthly samples, then this could be influenced by the pronounced seasonality of insect emergence. Directional change, if present, would then be visible only in regressions with yearly time lags. A detailed description of the method can be found in [23]. Euclidian distance among true fly communities at sites in different time intervals was calculated from dipteran family abundance data [30] to assess the temporal dynamics of dipteran communities within sites. Prior to analysis, monthly samples from each site were pooled (cumulative value of six pyramid traps per site) and abundance data were log-transformed.

Dipteran community change point models were calculated using the mcp-package, Regression with Multiple Change Points in R. In the mcp-package, regressions are done between generalized and hierarchical linear segments using Bayesian inference with a pre-set number of change points [31]. The regression was defined as a two-segment model for all sites (one change point), with local diversity (Shannon index) on the “response” axis (y) and months of sampling on the “time” axis (x).

Dipteran taxa that are potential indicators of time periods with different discharge regimes at each site were assessed using the multilevel pattern analysis in the indicspecies package in R. This package functions to assess the strength and statistical significance of the relationship between taxa occurrence/abundance and groups of samples, and as such, it gives a strong emphasis on rare species in comparison to other analysis biased towards dominant taxa. [32]. Samples were grouped in years determined by the GLMM as those with “normal”, “low”, or “high” discharge. Statistical significance values were set at *p* < 0.05.

## 3. Results

After a GLMM was conducted and “normal” discharge variability was calculated, it was proven that discharge was significantly lower in the drought period of 2011/2012 for all three longitudinally positioned sites: spring; upper tufa barrier, lower tufa barrier (Table 1 and Supplementary Material). In addition to the severe drought in 2011/2012, the year 2007 was also determined as a year with significantly lower discharge values, and 2014 was determined as a year of significantly higher discharge values for all sites (Supplementary Material). Spearman’s correlation coefficient showed no significant relationship when comparing water temperature with minimum, maximum, or average discharge rates.

**Figure 2:**
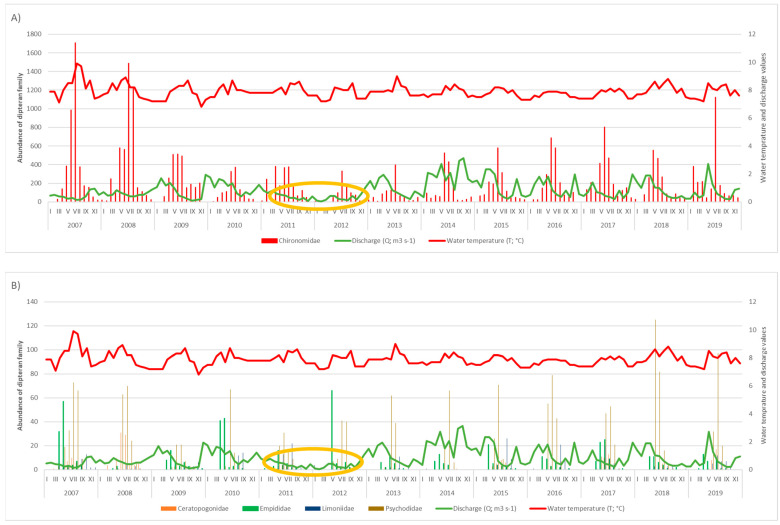

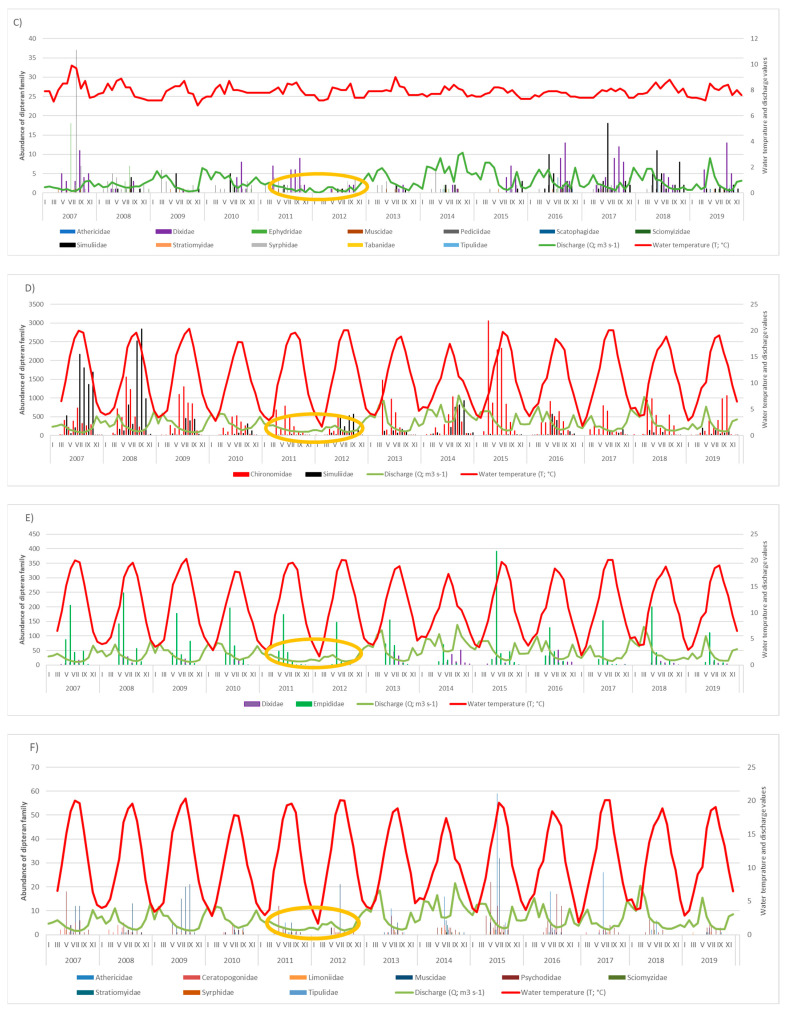

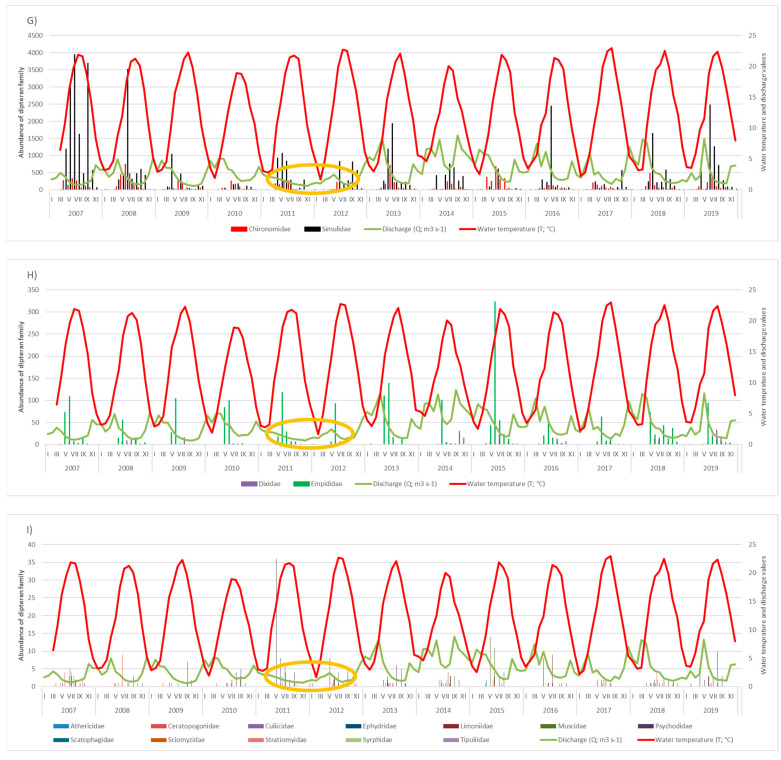
Aquatic true fly emergence patterns displayed in dipteran family abundance as columns in three habitat types from 2007 to 2019: (**A**) Dominant families (Chironomidae); (**B**) moderately abundant (Ceratopogonidae, Empididae, Limoniidae, and Psychodidae); and (**C**) rare (other) families of the spring habitat (IBR = Spring of Bijela Rijeka). Water temperature and discharge are presented as solid lines. The discharge values from the great drought event of 2011/2012 are marked as a yellow ellipse. (**D**) Dominant families (Chironomidae and Simulidae); (**E**) moderately abundant (Dixidae and Empididae); and (**F**) rare (other) families of the Upper tufa barrier (BL = Tufa barrier Labudovac). Water temperature and discharge are presented as solid lines. The discharge values from the great drought event of 2011/2012 are marked as a yellow ellipse. (**G**) Dominant families (Chironomidae and Simulidae); (**H**) moderately abundant (Empididae); and (**I**) rare (other) families of the Lower tufa barrier (BKM = Tufa barrier Kozjak-Milanovac). Water temperature and discharge are presented as solid lines. The discharge values from the great drought event of 2011/2012 are marked as a yellow ellipse.

The dynamics of aquatic true fly family abundances with regard to water discharge and temperature values were plotted over time for all three sites (Figure 2). The temperature values remained stable (seasonally) even throughout the drought period (2011/2012), whereas significant differences in discharge were evident as discharge varied stochastically over time.

A stochastic regression model simulation was produced when Euclidian distance values were plotted against monthly time lags for all three habitat types (Supplementary Material, Figure S1). The results in the regressions were not significant, with poor fits to the predicted regression lines (R^2^ < 0.008) and slopes approaching zero for all three habitat types.

**Figure 3:**
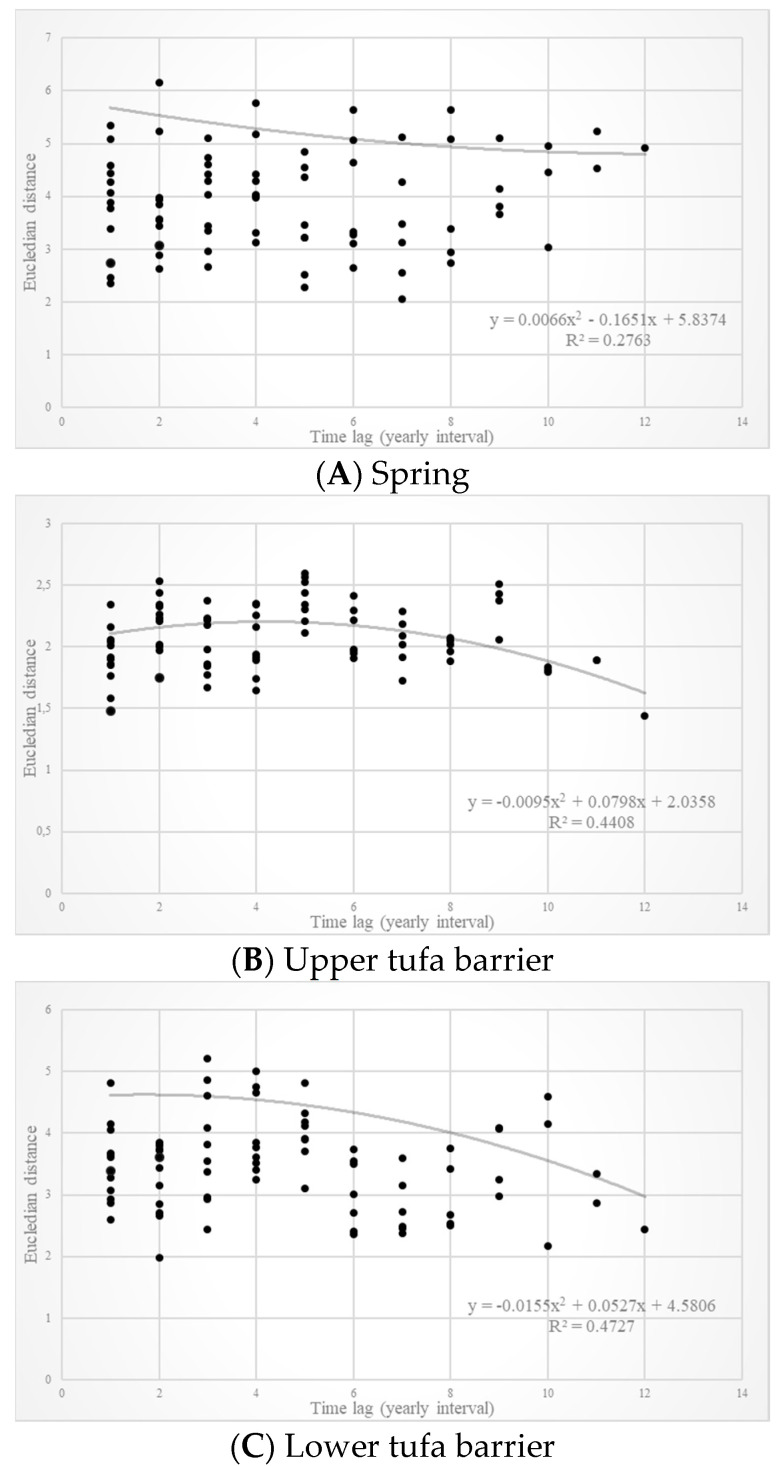
Euclidian distance triangular resemblance matrix of dipteran communities over time regression with monthly yearly lags (points represent communities compared among themselves with increasing time steps). The plot shows differences between dipteran community dynamics with 18 true fly families over 13 years for three longitudinally connected sites of the Plitvice Lakes NP: (**A**) Spring (IBR = Spring of Bijela Rijeka River), (**B**) Upper tufa barrier (BL = Tufa barrier Labudovac), (**C**) Lower tufa barrier (BKM = Tufa barrier Kozjak-Milanovac).

When Euclidian distance values between samples were plotted against yearly time lags, different trends were seen for the different habitats along the longitudinal gradient (Figure 3). Spring habitats showed less variation, with higher R^2^ values when compared to monthly intervals, though the regression slope was near zero (y = 0.0066x^2^ − 0.1651x + 5.8374; R^2^ = 0.2763). The ordination of compositional trends for the upper (y = −0.0095x^2^ + 0.0798x + 2.0358; R^2^ = 0.4408) and lower (y = −0.0155x^2^ + 0.0527 + 4.5806; R^2^ = 0.4727) tufa barrier clearly showed directional change. The short first parts of the regressions of the upper and lower tufa barrier show directional change away from the original communities followed by a possible recovery phase.

### 3.1. Spring

The change point analysis in the spring site showed a post-drought change (i.e., 2nd normal discharge period; cp_1 = 76.80 months ≈ May 2013, Figure 4A). The indicator species analysis detected Syrphidae (d = 0.368; *p* = 0.029) as an indicator of two time periods: 1st and 2nd low discharge period. Ephydridae were found as indicators of three time periods (d = 0.461; *p* = 0. 029): 1st low, 1st normal, and 1st high discharge period, and Simuliidae were found as indicators of 1st, 2^nd^, and 3rd normal and 1st high discharge periods (d = 0.554; *p* = 0. 042).

### 3.2. Upper Tufa Barrier

The change point analysis in the upper tufa barrier also showed a change point in the first month of the year post-drought (i.e., 2nd normal discharge period; cp_1 = 72.41 months ≈ January 2013, Figure 4B). The indicator species analysis detected Tipulidae (d = 0.333; *p* = 0.046) as indicators of the 2nd normal discharge period. Dixidae were found as indicators of all periods with the exception of the drought period, that is, the 2nd low discharge period (d = 0.679; *p* = 0. 004).

### 3.3. Lower Tufa Barrier

The change point analysis in the lower tufa barrier detected a change point in the 1st normal flow period (cp_1 = 35.39 months ≈ December 2009, Figure 4C). The indicator species analysis calculated no indicator taxa for all the normal discharge periods, whereas Ceratopogonidae were found to be indicators of the 1st low discharge period (d = 0.501, *p* = 0.008), and Muscidae were found to be indicators in the 1st and 2nd low discharge period, and the 1st high discharge period (d = 0.501, *p* = 0.019).

## 4. Discussion

The great drought of 2011/2012 [16] caused severe precipitation loss, and, as expected, discharge was significantly decreased at all three study sites to about half that of non-drought years in both the whole year cycle, and in the emergence season. The lack of water temperature differences is also notable, indicating that this ecosystem is predominantly ground-water fed, relying on, what hydrologists refer to as “old water” for the majority of discharge, as opposed to “new” or surface water [33,34]. It is also argued that perennial lotic ecosystems usually maintain their flow by being ground-water fed [33]. This, in turn, means that, although water temperature did not rise in parallel with the decrease in water discharge, a great strain was put on aquifers [18] supporting the predominantly ground-water-fed system with potentially long-term and unforeseeable consequences. An additional low discharge period was detected in the first year (2007) of the study, but lasted for a shorter time and with lower magnitudes of discharge loss for all determined sites. The longitudinal connection between sites was again shown in 2014, where all sites presented significantly higher discharge values.

As predicted, the analysis of the monthly time lags against the Euclidean distances showed high variation and little to no slope for the regression. This is most likely a result of pronounced natural, cyclic, or seasonal changes in community emergence patterns for all analyzed sites as explained in the Materials and Methods section and in [23].

### 4.1. Spring

The analysis of the yearly time lags against the Euclidean distances of the spring habitat showed less variation compared to monthly analysis, with higher R^2^ values when compared to monthly intervals, but still, a regression slope close to zero indicated community stability. However, multiple change point regression models detected community change corresponding with the end of the drought (i.e., the start of the 2nd normal discharge period) showing changes in community structure in the post-drought period (i.e., 2nd normal discharge period). This is likely due to gradual community shifts in terms of lower abundances of drought- (or low flow) resistant taxa in the post-drought period when the normal flow was restored. More precisely, the indicators species analysis showed that significant changes in community structure occurred in this period, and Ephydridae were considered an indicator taxa of the 1st low, 1st normal, and 1st high discharge period, despite their low abundance. This inconsistency in association with different discharge periods, that is, regimes may indicate tolerance to discharge oscillations or could contribute to their overall low abundances which is in concordance with [35] who stated that Ephydridae in karstic springs when present are usually not abundant. A much clearer picture was painted by the other two indicator taxa determined for the spring site. Syrphidae were highlighted as an indicator taxon of both the 1st and 2nd low discharge periods. It is possible that they are solely collected in low discharge periods because their predatory larva during this time when their habitat is restricted increases their overall density and, therefore, the likelihood that they are collected [17,21,36]. The opposite pattern was observed with the family Simuliide which was linked to all normal- and high-flow periods. The larvae of this family are sessile filtrators, heavily dependent on flow regime for feeding. These shifts in indicator taxa are expected since springs are usually considered to be among the most vulnerable habitats to climate change [13,37,38].

### 4.2. Tufa Barriers

The analysis of the yearly time lags against the Euclidean distances of the upper and lower tufa barriers indicates a non-linear trend with a directional change, corresponding to approximately the first four years of the studied period, meaning that the directional change is most likely due to both low discharge periods. A downward trend following this directional change could be interpreted as possible convergence (community recovery) after the 2nd and more extreme low-discharge period, as seen in the example of lake recovery after an acidification experiment by [23]. However, here the results are inconclusive, as the initial sampling year was also characterized by somewhat lower discharge rates.

In the upper tufa barrier especially, this analysis was confirmed by the multiple change point analysis that showed directional change influenced by 2nd and more extreme low discharge period and gave an indication of community recovery (in terms of regained diversity values) in the later years of sampling. An indicator taxon of the upper tufa barrier was Dixidae, a resilient group present in all periods except the 2nd low discharge period, meaning that they have a strong capacity to recover from no flow disturbance [19]. This is not surprising since they are filter-feeders in the larval phase and are closely linked with water discharge, which when higher, brings more seston particles to the tufa barriers [4,39] and consequently more potential food for Dixidae larvae. The period after the 2nd, more extreme, low discharge period was associated with Tipulidae as an indicator group. Most Tipulidae larvae are usually terrestrial or semiaquatic [40], and during the drought period, more potential microhabitats (both terrestrial and aquatic) could have been created for Tipulidae, thereby increasing their abundance in comparison with periods having normal and later higher water discharge levels.

Further downstream at the second tufa barrier, no taxa were found to be indicators of all three normal discharge periods. This is a possible result of greater dipteran family diversity (i.e., higher values of Shannon index) found in comparison to the other two upstream sites that would indicate more evenness in taxa distribution and, hence, no indicator taxa that stand out. Resistant taxa, namely Muscidae, were interestingly, associated with all extreme discharge periods, both low and high, thus exhibiting potential tolerance to discharge oscillation, but also an adaptation to living in the specific habitats of the tufa berries [9]. Here Ceratopogonidae were found associated with lower discharge periods, but only the 1st, more mild low discharge period, making the idea of Ceratopogonid tolerance level to low discharge still too vague to interpret. Although the change point in dipteran diversity was detected prior to the start of the 2nd, more extreme drought period, local diversity values were very low in the second part of this period and remained this way until the end of the study period with no natural variation, indicating directional change and diminished local diversity in the lower tufa barrier. In addition, the authors acknowledge that family-based interpretations of local change in dipteran community structure may hide behind the variation of specific species with various life histories. However, the trends and responses of some families to discharge, found in our research undoubtedly indicate changes in the composition of communities as a result of extreme discharge.

As [41] stated, disturbance history is as important as current disturbance is in structuring communities with particular functional features, for example, affecting one functional feeding group more than others [42], or favoring specific taxa with desiccation-resistance traits [43]. In this study, low discharge favored one group of insects with resilience to low-flow conditions. The abundance of dipterans with these traits in these communities could make the aquatic insect community sensitive to other different disturbances such as flooding and could cause even greater shifts in community composition. The same is possible for opposite scenarios. Invasive and/or pest species are also a major threat as they could proliferate in the weakened community [44]. Unfortunately, climate modelling predictions [15] indicate that further decreases in precipitation are to be expected in the study region during spring, summer, and autumn.

The patterns of directional change and community recovery detected in this study period are only visible thanks to many years of research efforts. As the issue of climate change is omnipresent and community responses are highly versatile and complex, the need for more long-term research such as [45] cannot be overstressed.

## 5. Conclusions

The dipteran community, because of its great diversity and pronounced seasonality, shows high natural variability in both abundance and structure. However, the significant shifts in community composition resulting from extreme discharge regimes show how even this highly variable community can clearly indicate environmental change and/or stress, but the patterns are only made visible after long-term data analysis.

## Figures and Tables

**Figure 4:**
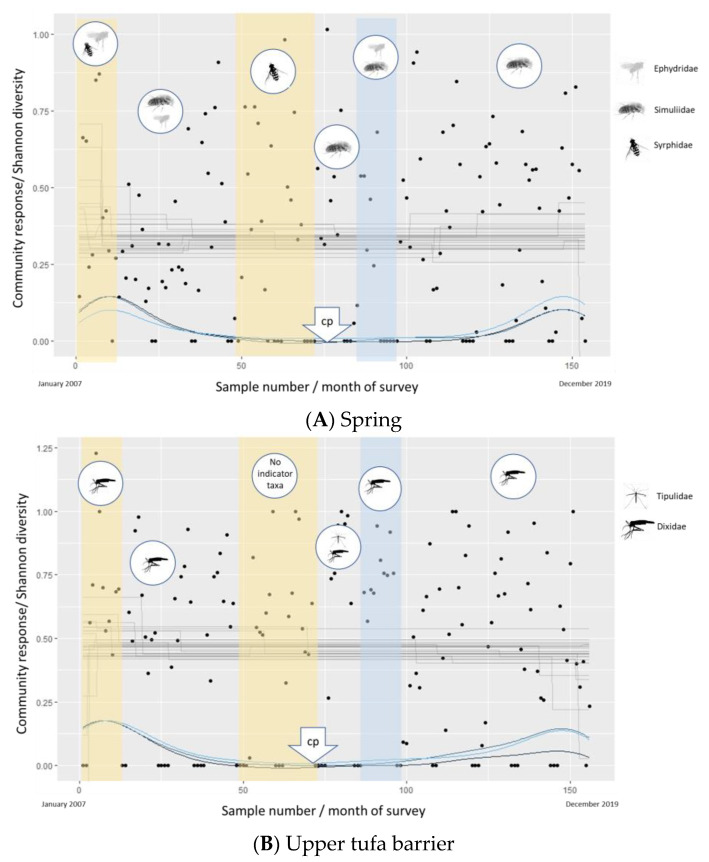

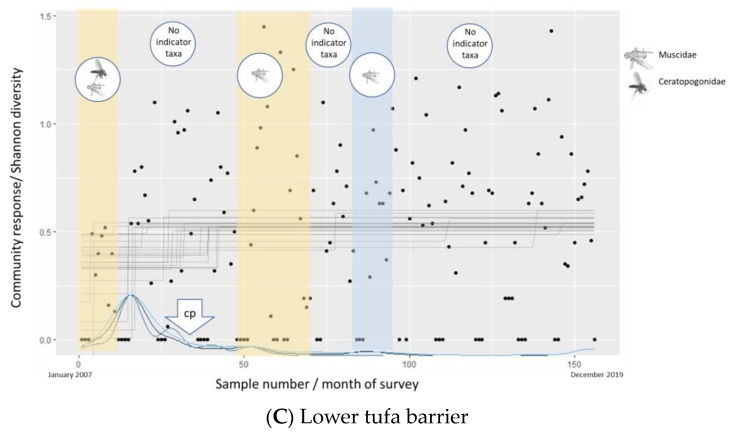
Changepoint model (cp = calculated change point) based on dipteran community response (Shannon index, *y*-axis) over a 13-year period (*x*-axis). The two low discharge periods (2007 and 2011–2012) determined with a generalized linear mixed model are shown in yellow shading, whereas one high discharge period (2014) is shown in blue shading. Grey lines show 25 draws from the posterior distribution of the mean community response. The posterior distribution for the change point is shown in dark blue on the *x*-axis, and the kernel density of replicated datasets produced by the fitted model is shown as a light blue line. Indicator taxa significantly linked to specific sites: (**A**) Spring, (**B**) Upper tufa barrier, (**C**) Lower tufa barrier; and periods: 1st Low, 1st Normal, 2nd Low (drought), 2nd Normal, 1st High, and 3rd Normal discharge period are presented as circles.

**Table 1:** Characteristics of the sampling sites. IBR–Spring of Bijela Rijeka, BL–Upper (upstream) tufa barrier Labudovac, BKM–Lower (downstream) tufa barrier Kozjak-Milanovac. Temperature and discharge values are shown separately for the drought period (2011/2012) and the rest of the research period: 2007–2011 and 2013–2019. Min–minimum; SD–standard deviation; Max–maximum; CV–coefficient of variance (SD/average) of the values within a given period.

Site		IBR	BL	BKM
Latitude		N 44°50″05′	N 44°52″17′	N 44°53″39′
Longitude		E 15°33″43′	E 15°35″59′	E 15°36″32′
Longitudinal position/habitat type in karst barrage lake ecosystem		Spring	Upper barrier	Lower barrier
Altitude (m)		720	630	546
Substrate		Pebbles and sand, Macrophytes, Moss	Pebbles, Moss on tufa, Tufa with detritus	Pebbles, Moss on tufa, Tufa with detritus, Silt
Water temperature (°C) without 2011/2012	Min	6.8	1.848233871	1.91925
	Average ± SD	7.884 ± 0.48	10.900 ± 5.45	12.296 ± 6.53
	CV	6%	50%	53%
	Max	9.9	20.33	22.95
Water temperature (°C) in 2011/2012	Min	7.2	1.663	1.673
	Average ± SD	7.846 ± 0.48	11.078 ± 5.45	12.603 ± 6.53
	CV	5%	57%	58%
	Max	8.6	20.063	22.683
Water temperature (°C) in vegetative season (April–October) without 2011/2012	Min	7.2	8.35	8.18
	Average ± SD	8.117 ± 0.45	14.655 ± 3.62	16.776 ± 4.17
	CV	6%	25%	25%
	Max	9.9	20.33	22.95
Water temperature (°C) in vegetative season (April–October) 2011/2012	Min	7.3	9.167	9.666
	Average ± SD	8.064 ± 0.39	15.629 ± 3.85	17.721 ± 4.39
	CV	5%	25%	25%
	Max	8.6	20.063	22.683
Discharge (m^3^/s) without 2011/2012	Min	0.001	0.495	0.613
	Average ± SD	0.547 ± 0.42	2.464 ± 1.893	3.211 ± 2.06
	CV	77%	77%	64%
	Max	2.83	15.6	29.8
Discharge (m^3^/s) in 2011/2012	Min	0.011	0.525	0.625
	Average ± SD	0.225 ± 0.201	1.349 ± 0.794	1.595 ± 0.845
	CV	89%	59%	53%
	Max	0.909	6.36	7.23
Discharge (m^3^/s) in vegetative season (April–October) without 2011/2012	Min	0.001	0.495	0.6781
	Average ± SD	0.502 ± 0.43	2.008 ± 1.79	2.688 ± 2.03
	CV	85%	89%	76%
	Max	2.83	15.6	8.794
Discharge (m^3^/s) in vegetative season (April–October) 2011/2012	Min	0.033	0.525	0.776
	Average ± SD	0.190 ± 0.13	1.077 ± 0.47	1.354 ± 0.51
	CV	70%	43%	38%
	Max	0.578	3.6	2.461

## Data Availability

The data presented in this study are available as appendix of this article and on request from the corresponding authors.

## Supplementary Materials

The following supporting information can be downloaded at: https://www.mdpi.com/article/10.3390/biology12040590/s1, Figure S1: Euclidian distance triangular resemblance matrix of dipteran communities over time regression with monthly time-lags (points represent communities compared among themselves with increasing time steps). Plot shows seasonal, non-directional dipteran community dynamics with 18 aquatic true fly families over 156 time steps (12 months × 13 years) for three longitudinally connected sites: (A) Spring (IBR = Spring of Bijela Rijeka River), (B) Upper (upstream) tufa barrier (BL = Tufa barrier Labudovac), (C) Lower (downstream) tufa barrier (BKM = Tufa barrier Kozjak-Milanovac). Different shades of grey are plotted for visualization purposes only, and Excel table: SuppInfo_Diptera_Abundance.
