# Supplementary material for: Defining Patterns and Rates of Natural vs. Drought Driven Aquatic Community Variability Indicates the Ongoing Need for Long Term Ecological Research
Source: Biology (Basel). 2023 Apr 12;12(4):590. doi: 10.3390/biology12040590 (PMC10136097; doi:10.3390/biology12040590)
Supplement: Supplementary file 1 [file biology-12-00590-s001.zip › Supplementary Figure S1.pdf]

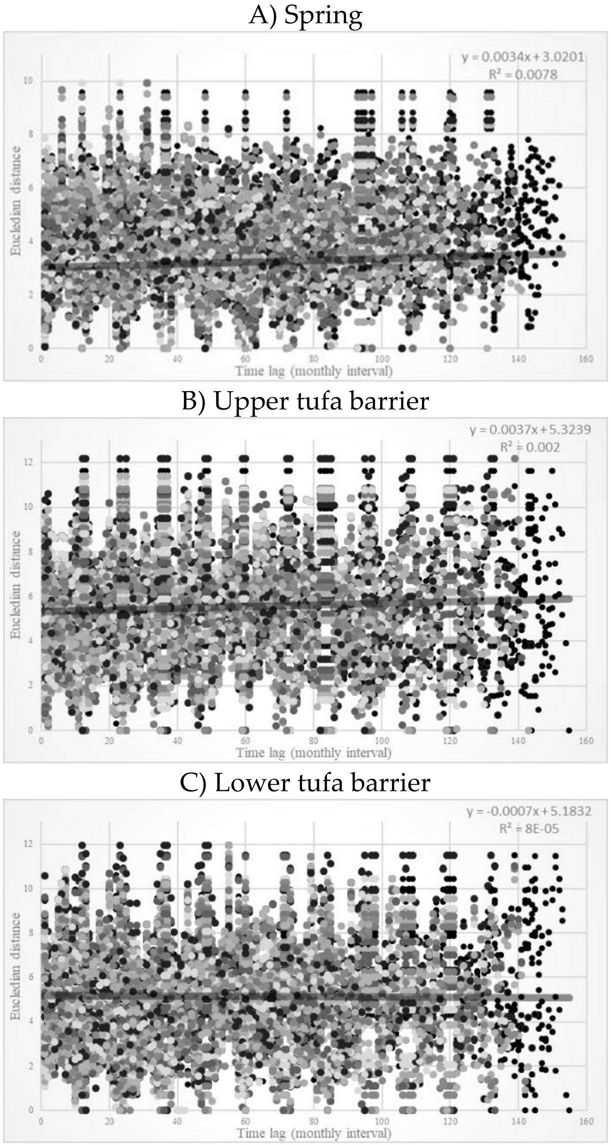

Figure S1. Euclidian distance triangular resemblance matrix of dipteran communities over time regression with monthly time-lags (points represent communities compared among themselves with increasing time steps). Plot shows seasonal, non-directional dipteran community dynamics with 18 aquatic true fly families over 156 time steps (12 months x 13 years) for three longitudinally connected sites: (**A**) Spring (IBR = Spring of Bijela Rijeka River), (**B**) Upper (upstream) tufa barrier (BL = Tufa barrier Labudovac), (**C**) Lower (downstream) tufa barrier (BKM = Tufa barrier Kozjak-Milanovac). Different shades of grey are plotted for visualization purposes only. .
